# Evaluation of the Cytological Changes of the Oral Mucosa Among Smokers in Al Madinah Al Munawara Using Argyrophilic Nucleolar Organizer Region (AgNOR) Counts and Papanicolaus Stain

**DOI:** 10.7759/cureus.39367

**Published:** 2023-05-23

**Authors:** Faris M Altom, Ghaidaa Y Bedair, Eman A Eysawi, Dalya K Hammoudah, Lina A Khoja, Rahaf A Yaseen, Ghazal M Sabooni, Zainah A Al Qahtani

**Affiliations:** 1 College of Medicine, Al-Rayan Colleges, Madinah, SAU; 2 Family Medicine, Medical Center Health System, Madinah, SAU

**Keywords:** agnor, pap, smokers, oral mucosal, cytological changes

## Abstract

Objective

To evaluate the cytological changes of the oral mucosa among smokers using Argyrophilic nucleolar organizer region (AgNOR) counts and Papanicolaou (Pap) staining.

Methodology

The oral mucosal exfoliate smears of 500 individuals (200 nonsmokers and 300 smokers) aged between 18 and 80 years were prepared in Al Madinah. The AgNOR count and Pap stain were used to generate a cytogenic smear to assess the presence of cytological changes suggestive of atypia, inflammation, dysplasia, keratinization, and proliferative activity of epithelial cells.

Results

Smokers have a considerably higher number of AgNORs per nucleus than nonsmokers (1.99 3.53 vs. 0.42 1.22). There were inflammatory changes in 127 (42.3%) of the cases and 40 (20%) of the controls. Multinucleated cells and atypia were found in 33 (11%) and 14 (4.5%) of the cases but not in the controls. The results indicate higher proliferative activity in smoking patients compared to nonsmoking patients, even in the absence of clinical lesions.

Conclusion

To detect the effects of smoking on the oral mucosa, Pap staining alone is insufficient. Combining Pap staining with the AgNOR technique produces the desired results.

## Introduction

Smoking is presently the most preventable cause of disease, and one of the most important risk factors for the emergence of cancer in many organs on a worldwide scale is mortality. In light of the plethora of alterations that tobacco can cause, it is necessary to closely monitor patients who smoke [1]. Oral squamous cell carcinomas (OSCCs) are currently ranked sixth in the world, and the five-year survival rate for cancer is dismal unless diagnosed in the early phases [2].

Therefore, there is a need to encourage the early detection of oral malignancies. The gold standard is a biopsy, which is performed only when lesions become symptomatic, i.e., in the late or advanced stage. Microscopically distinguishing malignant from benign abnormalities is fraught with difficulty, and routine histopathological techniques do not reveal all diagnostic and prognostically significant characteristics [3].

Therefore, it is imperative to develop adjunct procedures that accurately and promptly predict malignancy progression. The significance of the nucleus in tumor pathogenesis has undergone a paradigm shift following the discovery of a correlation between nucleolar function, size, and cell doubling time in human cancer cell lines.

In order to discover and track the earliest cellular changes, exfoliative cytology may prove useful, reflecting the crucial function of the nucleus in the regulation of cell proliferation and protein synthesis. Ki-67's estimates of the proliferation rate and the proportions of S-phase and mitotic cells all correlate with Argyrophilic nucleolar organizer region (AgNOR). Consequently, the NOR are loops of ribosomal DNA present in the nucleoli of cells on the short limbs of acrocentric chromosomes 13, 14, 15, 21, and 22 [4].

Under a light microscope, the interphasic NORs are easy to see because the interphasic acidic proteins are stained by a silver reaction NOR (RNA polymerase 1 upstream binding factor, topoisomerase 1, nucleolin, fibrillin, C23 protein, and B23 protein). Small black specks scattered around the nucleolus after silver staining can be used to identify AgNORs. Quantitatively, AgNORs are a marker of cell proliferation based on their abundance in the nucleus [4]. It is a complementary diagnostic technique that has many advantages, including quick and easy execution, low cost, diagnostic safety, efficacy, and noninvasiveness. It can also be used repeatedly. Staining with Papanicolaou (Pap) is only useful for screening. It lacks diagnostic value. Only those who are susceptible to dysplasia or cancer are recognized. AgNOR counts can serve as an indicator of premalignant or malignant oral mucosal alterations.

The goal of the current study was to assess the cytological changes of the oral mucosa among smokers using AgNOR counts and Pap staining.

## Materials and methods

Study design

This was a case-control study conducted in Madinah that was carried out from August 2022 to March 2023.

Sample collection

Using a wooden spatula, buccal mucosal exfoliative samples were obtained from 500 participants (300 smokers and 200 nonsmokers) aged between 18 and 80 years. The subsequent inclusion criteria were implemented: non-alcohol use, absence of oral lesions, and absence of malignant or benign oral neoplasms in the past or present. Analyses were conducted on both smokers and nonsmokers.

Those who smoked 20 or more cigarettes per day were classified as smokers and those who smoked < 20 cigarettes per day were considered non-smokers [5]. Only filtered cigarette smokers only were considered for this study. The researchers left out pipe smokers and other tobacco consumers because of the potential for concentration change, which could have distinct effects on oral mucosal cells, and other systemic consequences [6]. People who had never smoked were considered non-smokers. Pap and AgNOR stains were used to color the cytologic samples. and viewed at magnifications of 40 and 100, respectively.

AgNOR staining

The air-dried smudges were discolored. The freshly prepared working solution was prepared by combining two volumes of a 50% aqueous silver nitrate solution with one volume of 2% gelatin in a 1% formic acid solution. Before analysis, all smears were left in a dark, room-temperature silver solution for 30 minutes [7]. Cytopathologists examined and interpreted the silver-stained cells at 10x and 40x magnification using a light microscope. AgNORs were enumerated in the nuclei of the first 50 non-overlapping, inner-layer nucleated epithelial cells. Under a light microscope, NORs were directly counted based on the parameters established by Crocker et al., i.e., well-defined black dots in the nucleus were counted, and aggregations (overlapping or fused black dots) were deemed a single structure [8].

Papanicolaou staining (Pap) technique

All around the world, the Pap test has been used to detect precancerous and cancerous tumors. After 15 minutes in 95% ethanol, the smears were fixed. rinsed in tap water, staining with Harris hematoxylin for one to three minutes, rinsing with running water, dipping in 95% ethanol, staining with eosin azure for 2-2.5 minutes, two 10-second dips in 95% ethanol, one minute in 100% ethanol, and two 10-second dips in xylene to remove the stain, and finally mounted with dibutyl phthalate xylene [9]. Pap-stained smears were identified at 40x.

Cytological changes suggestive of inflammation, dysplasia, keratinization, and proliferative activity of epithelial cells, Epithelial atypia can be considered in the presence of one or more of the following features: hyperchromatism, an increased nuclear-to-cytoplasmic ratio accompanied by nuclear enlargement, clumping of chromatin characterized by moderately prominent nucleation and irregular nuclear borders, bi- or multinucleation, increased keratinization and scantiness of the cytoplasm, and variations in cell size and shape.

Data management

The Statistical Package for Social Science (SPSS), version 21.0 (IBM Corp., Armonk, NY), was used for data coding, entry, and statistical analysis. With a chi-squared for categorical variables (P < 0.05), the data is deemed significant.

Ethical approval

The study was approved by the Medical Ethics Committee of Al-Rayan Colleges (ethical approval code: H-03-M-122-024z).

## Results

In this study, the ages of 500 participants (300 smokers (case) and 200 non-smokers (control) ranged from 18 to 80 years with a mean age of 28 years. There were 1.39 males for every female present. Males and females had roughly equal representation across all age groups as indicated in Table 1.

**Table 1:** Distribution of age group by gender

Age groups	Gender	
	Male	Female
≤ 20 years	56	60
21-29	94	88
30-39	54	47
40-49	49	14
≥ 50	28	11
Total	280	220

**Figure 1:**
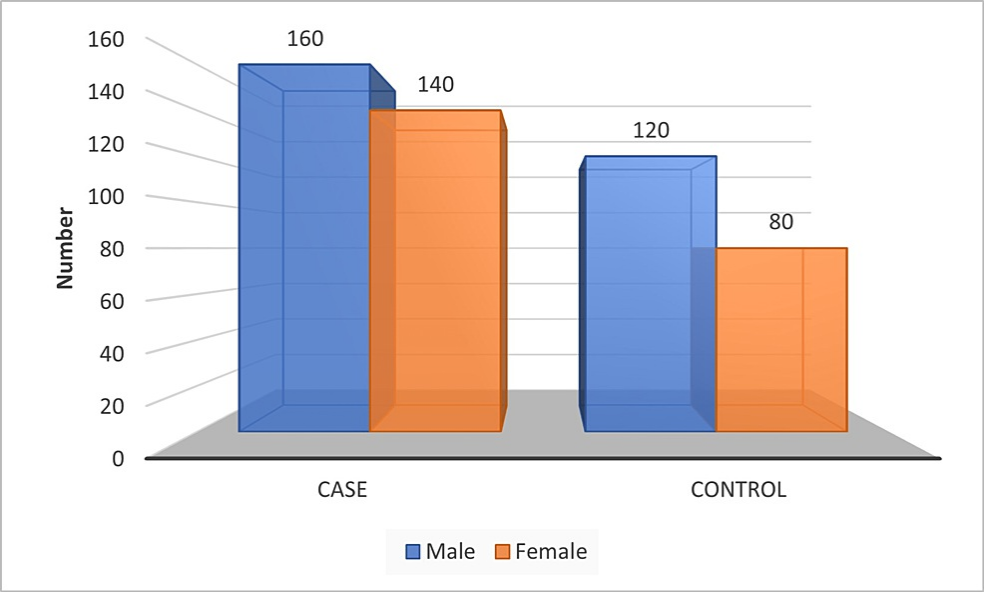
Distribution of the study population by gender

The distribution of the study population by gender is shown in Figure 1. Both cases and controls were more frequently seen among males as compared to female smokers (160 to 140 cases) and non-smokers (120 to 80 cases), respectively.

**Figure 2:**
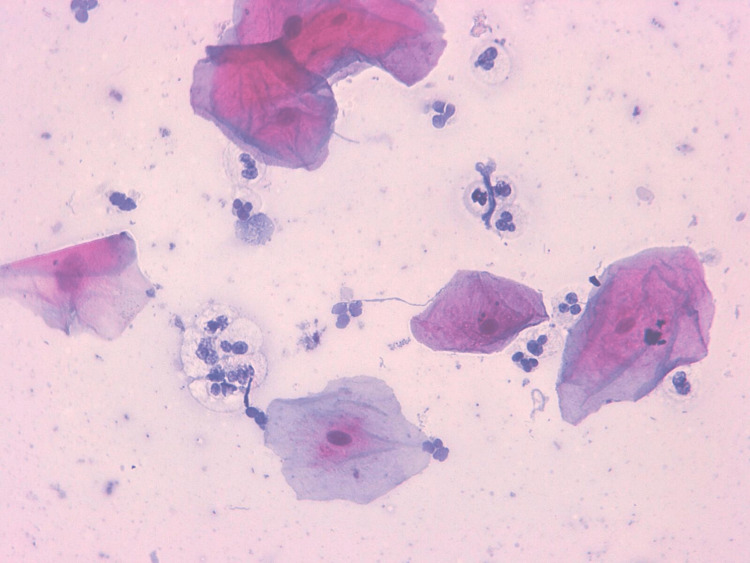
Buccal smear taken from an apparently healthy cigarette smoker The smear shows epithelium cells with inflammation. Papanicolaou (Pap) stain X40

**Figure 3:**
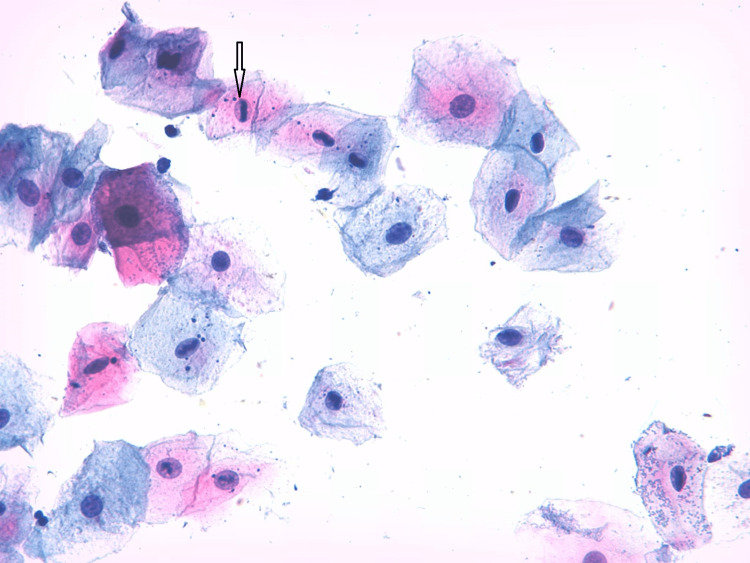
Buccal smear taken from an apparently healthy cigarette smoker The smear shows epithelium cells with atypia. Papanicolaou (Pap) stain X40

From 500 samples stained with Papanicolaou (Pap), inflammatory changes were detected in 42.3% of cases and 20% of controls; multinucleated and atypia were detected in 11 and 4.5 percent of cases, respectively, and not detected in controls, as shown in Table 2, Figure 2, and Figure 3.

**Table 2:** Statistical analysis of cytological changes by Pap stain Pap: Papanicolaou

group	Cells - binucleated or multinucleated	Inflammation	Infection	Atypia	p. value
smokers	33 (11%)	127 (42.3%)	39 (13%)	14 (4.50%)	0.023
nonsmokers	0 (0%)	40 (20%)	6 (3%)	0 (0%)	

**Figure 4:**
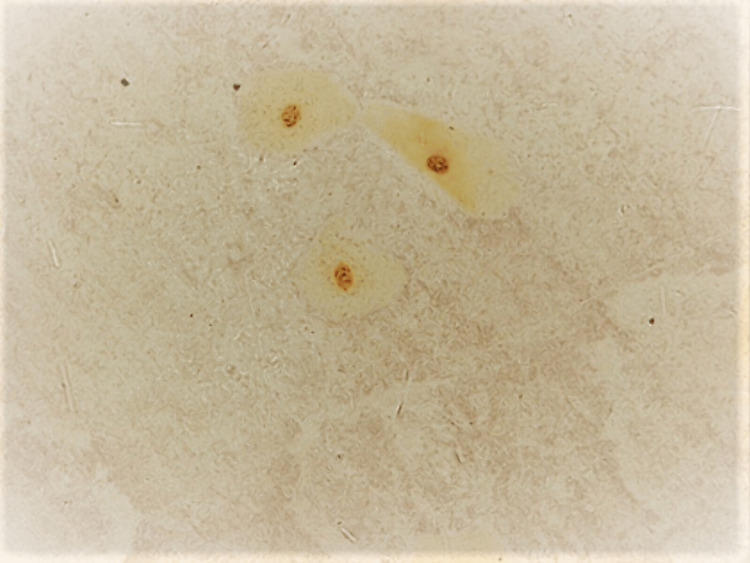
Buccal smear taken from an apparently healthy cigarette smoker The smear shows AgNOR dots per nucleus. Silver stain X40; AgNOR: Argyrophilic nucleolar organizer region

To evaluate the proliferative activity, the percentage of cells with more than three AgNORs per nucleus and the mean percentage of cells with more than three AgNORs per nucleus was higher in smokers (74.6%) than in nonsmokers (5.4%), with a significant difference (0.053) as shown in Table 3 and Figure 4. 

**Table 3:** Statistical analysis of AgNOR dots in the oral mucosa of the study population AgNOR: Argyrophilic nucleolar organizer region

AGNORS	No	Mean of AGNOR ± SD	Mean of % of cells with >3 AgNOR dots ± SD	p. value
Case	300	1.99 ± 3.53	74.6	0.053
Control	200	0.42 ± 1.22	5.4	

**Figure 5:**
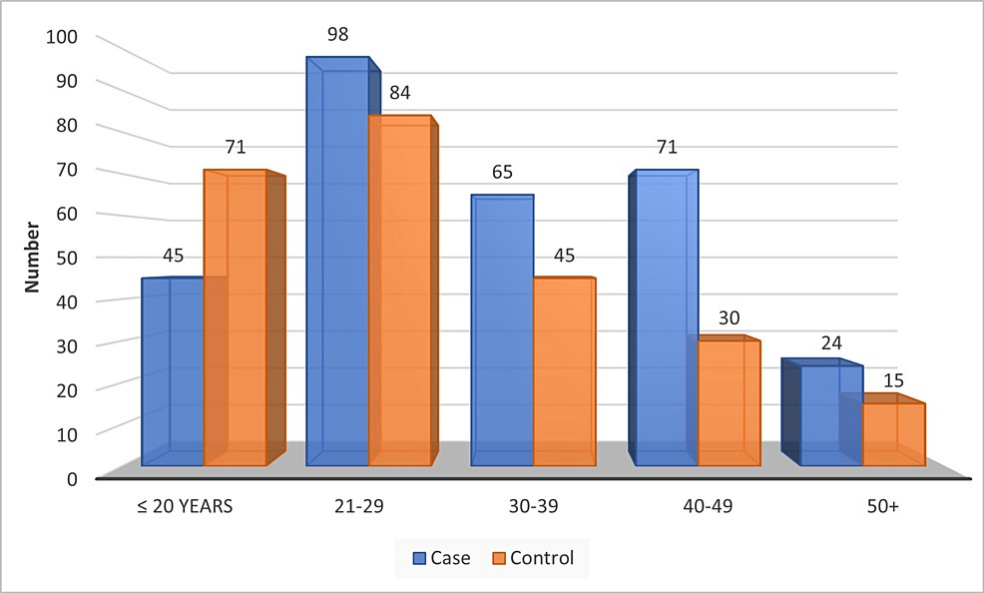
Distribution of age group by the study population

The majority of cases and controls were found in the age group of 21-29, followed by 40-49 in cases and 18-20 in controls, as shown in Figure 5.

## Discussion

The risk of oral cancer increases with the number of cigarettes smoked per day and the length of time a person smokes. Many people have experienced alterations to their oral mucosa as a direct result of their habit of smoking. Smoking has been linked to a wide variety of diseases, from superficial and easily treated lesions to more serious cancers of the oral mucous membranes [10].

This study provided conclusive evidence linking cytological alterations, AgNOR counts, and cigarette smoking. Oral mucosal epithelial cells proliferate more rapidly when exposed to heat and the chemical components of tobacco. Therefore, the risk of developing OSCC is greater for smokers. AgNOR staining allows the detection of this proliferation in the absence of clinical signs. Our data also showed that smokers have a higher average number of AgNOR per nuclear cell.

Cigarette smokers without oral lesions, like those in our study, have been successfully studied using the AgNOR method, which reveals higher rates of cellular proliferation in smokers as compared to nonsmokers. When studying the oral mucosal cells of smokers, Salehinejad et al. found a higher number of AgNORs per nucleus in smokers (3.6) compared to nonsmokers (1.96) [11]. Fontes et al. examined the AgNOR count of the tongue in cigarette smokers and non-smokers based on the number of cigarettes ingested daily and the length of smoking. It was discovered that tobacco consumers have a high risk of developing premalignant lesions [12].

Using the AgNOR and Pap techniques, Ahmed et al. analyzed the atypical cytological alterations in normally developing oral mucosa after exposure to tobacco, alcohol, spicy foods, and pepper. While the Pap method detected increased keratinization in 45% of smokers, 32.7% of pepper and hot meal consumers, 11.8% of alcoholics, and 3.7% of nonexposed individuals, we found that the mean number of AgNORs per nucleus was significantly higher in those who regularly smoked (3.68), drank (2.82), and consumed hot meals (2.28) [13].

In our investigation, a statistically significant difference between smokers and nonsmokers was found in the mean number of AgNORs per nucleus in 50 cells (1.99±3.53) and non-smokers (0.42±1.22). These values are comparable to those reported by Shah and Sethi and Orellana-Bustos et al. but differ from those obtained by Cancado et al., who observed a mean of 2.01 ± 0.13 in smokers [14-16]. This discrepancy may be attributable to the sample size and characteristics, as the latter authors defined smokers as those who had smoked 10-20 cigarettes per day for 10-50 years, whereas the inclusion criterion used in the present study was 10-20 cigarettes per day. In addition, neither the mean number of cigarettes nor the mean duration of smoking consumed daily was reported by the authors.

In mucosal cells of the buccal mucosa, smoking resulted in a considerably increased number of AgNORs per nucleus, showing that these cells are more proliferative. Sampaio HC hypothesized that smoking affects the proliferative activity of cells utilizing the histochemical AgNOR technique [17].

Even if there are no clinical symptoms, smokers have been found to have cytological changes like hyperchromaticity, an increased nuclear-cytoplasmic ratio, irregular nuclear borders, inflammation, multi or bi-nucleation, increased keratinization, and changes in the shape and/or size of the cells and nuclei. This may be a sign of epithelial changes in response to a physiochemical environment. In a similar study comparing smoking's effects on buccal epithelial cells in smokers and nonsmokers, Farhadi et al. found that smokers were more susceptible to nuclear alterations such as micronuclei formation and karyolysis frequency [18]. Even in the absence of clinical lesions, the results of this study indicate that smoking patients have greater proliferative activity than nonsmoking patients.

Limitations

The limitation of the present study is a small sample size, and it is unclear if smokers are at a higher risk of malignant transition to OSCC than nonsmokers. More study is needed to assess the detection of oral cancer utilizing the Pap stain and AgNOR technique on a larger sample size.

## Conclusions

The combination of Pap staining and the AgNOR method is a reliable indicator for measuring cigarette smoking's impacts on the oral mucosa. To find out, additional lengthy research with sizable sample sizes of cigarette smokers must be conducted.
